# Direct Visualization of Large‐Scale Intrinsic Atomic Lattice Structure and Its Collective Anisotropy in Air‐Sensitive Monolayer 1T’‐ WTe_2_

**DOI:** 10.1002/advs.202101563

**Published:** 2021-08-31

**Authors:** Kangdi Niu, Mouyi Weng, Songge Li, Zenglong Guo, Gang Wang, Mengjiao Han, Feng Pan, Junhao Lin

**Affiliations:** ^1^ Department of Physics Southern University of Science and Technology Shenzhen 518055 China; ^2^ Shenzhen Key Laboratory of Advanced Quantum Functional Materials and Devices Southern University of Science and Technology Shenzhen 518055 China; ^3^ School of Advanced Materials Peking University Shenzhen Graduate School Shenzhen 518055 China

**Keywords:** air‐sensitive 2D materials, anisotropic ripple, large‐scale atomic mapping, Te vacancy, WTe_2_ monolayer

## Abstract

Probing large‐scale intrinsic structure of air‐sensitive 2D materials with atomic resolution is so far challenging due to their rapid oxidization and contamination. Here, by keeping the whole experiment including growth, transfer, and characterizations in an interconnected atmosphere‐control environment, the large‐scale intact lattice structure of air‐sensitive monolayer 1T’‐WTe_2_ is directly visualized by atom‐resolved scanning transmission electron microscopy. Benefit from the large‐scale atomic mapping, collective lattice distortions are further unveiled due to the presence of anisotropic rippling, which propagates perpendicular to only one of the preferential lattice planes in the same WTe_2_ monolayer. Such anisotropic lattice rippling modulates the intrinsic point defect (Te vacancy) distribution, in which they aggregate at the constrictive inner side of the undulating structure, presumably due to the ripple‐induced asymmetric strain as elaborated by density functional theory. The results pave the way for atomic characterizations and defect engineering of air‐sensitive 2D layered materials.

## Graphical abstract

The large‐scale intrinsic lattice structure of air‐sensitive 1T’‐WTe_2_ monolayer is directly revealed by atomic scanning transmission electron microscopy imaging with dedicated sample protection. The collective thermal equilibrium lattice distortion, i.e., rippling, is found to be anisotropic and propagate only perpendicular to one of the preferential lattice planes, completely different than monolayer graphene and MoS_2_ with hexagonal symmetry.

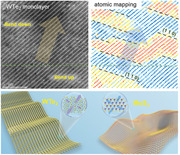

## 1 Introduction

The discovery of graphene has stimulated the widespread exploration of 2D layered materials,^[^ ^1^, ^2^, ^3^, ^4^ ^]^ which have significant potential in the applications of nanoelectronics, optoelectronics, and spintronics.^[^ ^5^, ^6^, ^7^, ^8^ ^]^ Emerging 2D materials exhibit promising physical and chemical properties that are quite different from their bulk counterparts, owing to the unique atomic thickness and tailored electronic structures in the reduced dimension.^[^ ^9^, ^10^, ^11^, ^12^ ^]^ As a representative emerging 2D material, 1T’ phase tungsten ditelluride (WTe_2_) is an type‐II Weyl semimetal^[^ ^13^, ^14^ ^]^ which has been demonstrated to maintain topologically protected electronic states, therefore a promising candidate for customized and programmable electronic devices as well as quantum spin Hall devices.^[^ ^15^, ^16^, ^17^ ^]^ Besides, 1T’ WTe_2_ have been reported to exhibit anisotropic Ising superconductivity, nonsaturating magnetoresistance, and tunable ferroelectricity,^[^ ^18^, ^19^, ^20^, ^21^, ^22^, ^23^, ^24^ ^]^ all of which are presumably associated to the exotic 1T’ lattice structure that holds low symmetry.

The fascinating physical properties in 2D WTe_2_ and other emerging 2D materials call for in‐depth studies on the local atomic structure of lattice and defects, which substantially affect the properties of the materials.^[^ ^25^, ^26^, ^27^, ^28^, ^29^ ^]^ Atomic resolution imaging techniques such as scanning transmission electron microscopy (STEM) is often used to study the lattice structure and surface morphology of regular 2D materials such as MoS_2_.^[^ ^30^, ^31^ ^]^ However, most of the emerging layered 2D materials suffer from high structural instability when exposing to the ambient conditions,^[^ ^32^, ^33^, ^34^, ^35^ ^]^ which severely damage the structural integrity during the STEM sample preparation. A typical example is 2D ferromagnet such as atomically thin CrI_3_, which can be mechanically exfoliated in the glove box.^[^ ^36^, ^37^ ^]^ However, its structural characterization at atomic resolution is rarely reported. This is also true for chemical vapor deposition (CVD) samples. Indeed, CVD‐grown monolayer WTe_2_ degrades in 5 min after fetched out from the furnace due to rapid oxidization and contamination in air,^[^ ^38^, ^39^, ^40^ ^]^ rendering a damaged sample for atomic STEM imaging.

The common method used to protect samples from degradation is encapsulation based on graphene^[^ ^41^, ^42^ ^]^ or hexagonal boron nitride,^[^ ^43^, ^44^, ^45^ ^]^ in order to prevent the interaction between the sample and the environmental oxygen. However, it should be noted that encapsulation through dry transfer method is hard to maintain a clean and sharp interface where contamination is easily introduced during the process, which hinders direct observation of the intrinsic lattice structure after encapsulation. Moreover, encapsulation increases the scattering events due to the irrelevant protection layer during atomic STEM imaging, adding more complexity in interpreting the atomic structure of the atom‐thin layer buried inside the capsulation. Developing a nondestructive and clean means which is compatible to atomic TEM/STEM imaging, therefore, is of high urgency for the atomic‐scale characterizations of the intrinsic lattice and defect structures in air‐sensitive 2D materials.

Herein, we develop a strategy to perverse the large‐scale intact lattice structure of air‐sensitive 2D materials which is compatible with atomic STEM imaging. As a proof of concept, we directly visualize the large‐scale intrinsic atomic lattice structure of air‐sensitive 1T’‐WTe_2_ monolayer grown by CVD method through Z‐contrast STEM imaging. The entire process, from the growth, transfer to STEM imaging, is operated in the inert gas environment inside a home built interconnected glove‐box system, entirely isolated from the ambient environment. Such setting ensures the surface cleanliness and structural integrity of the air‐sensitive 2D samples. Benefit from the large‐scale atomic imaging, we observe collective lattice distortions in continuous suspended 1T’ WTe_2_ monolayer due to the presence of intrinsic anisotropic rippling, which is verified by the biaxial tilted series of TEM electron diffraction patterns (DP). We find that the anisotropic lattice ripples have a dominating direction which is perpendicular to either crystal planes of (100), (110) or (1−10) depending on the initial sample conditions, whereas cannot coexist in the same monolayer. The bending curvature and propagation direction of the ripples can be determined by the distribution of the distorted W‐Te quadrilateral units. Such structural anisotropy stemmed from the anisotropic lattice of 1T’ phase WTe_2_ which lack of rotational symmetry, giving rise to diverse response and preferential tolerance to the strain along certain fixed directions. Moreover, the anisotropic rippling structure also leads to preferential formation of intrinsic Te vacancies in the constrictive inner side of the undulating structure, as elaborated by density functional theory (DFT) calculations. The electronic structure of the WTe_2_ monolayer is also regulated by the anisotropic rippling morphology, in which the spin–orbit‐coupling (SOC) induced band gap is calculated as 0.22 eV, larger than that of the flat case (0.18 eV). Our results pave the way for atomic scale study of surface morphology and intrinsic defect structure in air‐sensitive 2D materials and also offer new opportunity in anisotropic defect engineering.

## 2 Results and Discussion

**Figure 1:**
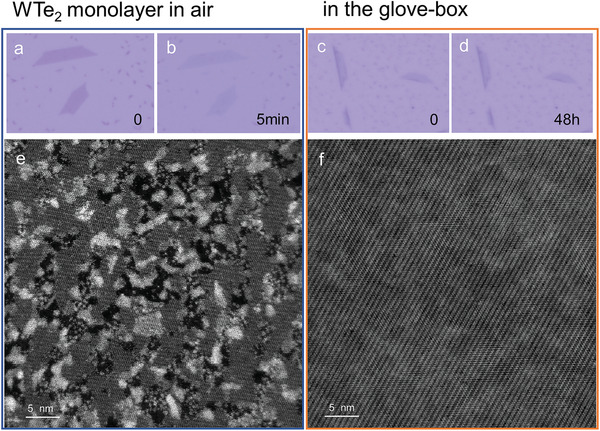
Optical and STEM imaging of air‐sensitive WTe_2_ monolayer. Optical images of 1T′‐WTe_2_ exposed in air a) right after the growth and b) after 5 min. A clear dimming of the contrast from a) purple to b) pale purple is visible. Optical images of 1T’‐WTe_2_ in the glove‐box c) right after the growth and d) after 48 h. No obvious change of contrast is visible. Large‐scale high‐angle annular dark field (HAADF) STEM images of large‐scale WTe_2_ monolayer prepared e) in air and f) in the inert environment of the glove‐box, respectively. The sample surface is deteriorated and fragmented due to the oxidation and contamination in air, while the large‐scale intact atomic lattice structure is clearly visible when the entire process is isolated with air.

1T′ phase WTe_2_ flakes are grown by CVD method (see the Experimental Section for detail) and the monolayer WTe_2_ flakes are verified by atomic force microscopy (AFM) (Figure S1, Supporting Information). Due to the metastability of air‐sensitive 1T’‐WTe_2_ monolayer, the surface structure is easily oxidized and degraded in minutes. The variation can be monitored intuitively via optical contrast.^[^ ^46^, ^47^ ^]^ When 1T’‐WTe_2_ monolayer is exposed to air, as shown in **Figure** **1**a,b, a significant fading of the contrast is visible within 5 min. Rapid transfer and atomic STEM imaging of the exposed WTe_2_ monolayer is performed right after the growth, where the total process is within one hour. Due to the aggressive oxidation of oxygen in the air, sensitive WTe_2_ monolayer is rapidly oxidized and not able to survive. Obviously, WTe_2_ monolayer severely degraded and collapsed with a huge number of oxidized nanoparticles and contaminants, breaking the periodicity of the lattice (Figure 1e). In a sharp contrast, the 1T’‐WTe_2_ monolayer under protection of inert environment exhibits no significant change of optical contrast over 48 h after growth (Figure 1c,d). To preserve the structural integrity in atomic STEM imaging, we conducted the entire experiment, including growth, transfer, and characterization, within the inert gas environment (home‐built glovebox connected system, see Figure S2, Supporting Information). An isopropanol assisted direct wetting transfer method was used, which is a common method to transfer a thin‐film sample onto the TEM grid. This method preserves the intrinsic nature of the sample to the greatest extent. The final TEM grid after transfer is loaded into TEM column by a vacuum transfer holder to isolate any air exposure. As shown in Figure 1f, the large‐scale intrinsic lattice structure of monolayer WTe_2_ can be clearly revealed at the atomic scale, which makes it possible to study the intrinsic lattice continuity and surface morphology of the 1T’‐WTe_2_ monolayer, which has lower symmetry than the 2H phase.

**Figure 2:**
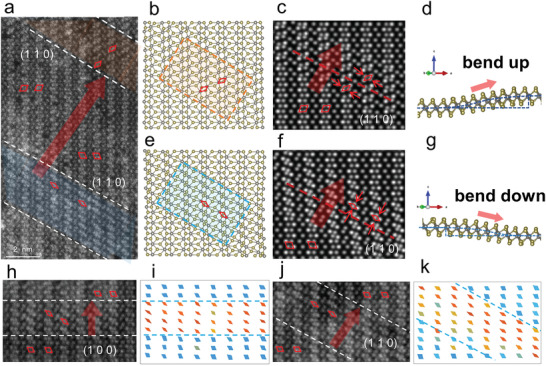
Atomic characterizations of the collective distorted lattice in continuous monolayer WTe_2_. a) HAADF‐STEM image of WTe_2_ monolayer. Corrugated areas are marked by orange and blue shades representing the ascending and descending sections of the ripple structure, respectively. The direction of the crystal plane (110) is marked by white dotted lines, which is also the boundary of the ripple structure. The arrows indicate the direction of the ripple. b,e) Atomic models of the marked orange and blue regions in (a), shown from top view. The deformed region and its bending direction can be clearly distinguished by the orientation of the distorted quadrilateral unit (marked by red diamonds), with b) the upward‐bending structure shaded in orange and e) the downward‐bending in blue, respectively. c,f) Corresponding STEM simulation images and d,g) atomic models from the side view. The ripple propagates dominantly perpendicular to the (110) crystal plane. h,j) HAADF‐STEM image of ripples propagating perpendicular to the (100) and (110) crystal planes. i,k) The spatial distribution mapping of distorted quadrilateral units in (h, j). The degree of distortion is reflected by the quadrilateral color changing from blue (less distortion) to red (severer distortion). The distribution of the distorted quadrilateral units is used as a key feature to distinguish the direction of ripple spread.

Benefit from the dedicated protection, the large‐scale atomic lattice structures of monolayer WTe_2_ are clearly observed, where some unusual chain‐like distorted structures are regularly spotted in clusters, totally different from the intrinsic WTe_2_ lattice structure. The existence of these distorted chain‐like structures is omnipresent in every flake we examined (Figure S3, Supporting Information). The distorted regions, as highlighted by the orange and blue shades, are found to be seamlessly embedded in the flat WTe_2_ monolayer lattice with gradually fluctuating patterns, as shown in **Figure** **2**a. Fast Fourier transformation of these regions did not show additional symmetry (Figure S4, Supporting Information). Combining the atomic STEM simulations, we discovered that these distorted regions are the ascending and descending regions of the fluctuating structure in monolayer WTe_2_. Figure 2b,e shows the projected atomic structure of the monolayer WTe_2_ when bending up and down, respectively (side views in Figure 2d,g). Due to the lattice deformation during bending, the projected quadrilateral unit (marked by red diamonds) formed by Te and W columns would be squashed depending on the bending directions as compared to the intrinsic flat region, as shown by the red arrows in Figure 2c,f. Such alignment of the distorted quadrilateral units is a key feature in determining the morphology of the ripple. Therefore, in Figure 2a, the highlighted shades represent the ascending and descending sections of the ripple structure, and together form a continuous ripple. By mapping the spacing of the squashed quadrilateral units, we can reconstruct the 3D model and further estimate the bending slope of the ripple as ≈0.2 (see details in Figure S5, Supporting Information).

Furthermore, the undulating direction can be determined by the distribution of the distorted quadrilateral units, which can be extracted for spatial distribution mapping. The cutoff boundary between the distorted and the intrinsic flat regions (Figure 2i,k) reveals undulating ripple direction. Figure 2h,j displays two bending structures undulating perpendicular to the direction of crystal plane (100) in Figure 2h and (110) in Figure 2j (in two different flakes), respectively. The sharp cutoff boundary along either (100) and (110) lattice planes suggest these collective lattice distortions, i.e., ripples in WTe_2_ monolayer, have highly anisotropic prorogation. It is straightforward to point out that due to lattice symmetry, such anisotropic ripples are also form perpendicular to the (1–10) crystal planes, as evidenced in atomic STEM images (Figure S6, Supporting Information). The results discussed above also apply to the WTe_2_ monolayer obtained by mechanical exfoliation, as shown in the Figure S7 in the Supporting Information, which confirm the intrinsic nature of these ripples.

**Figure 3:**
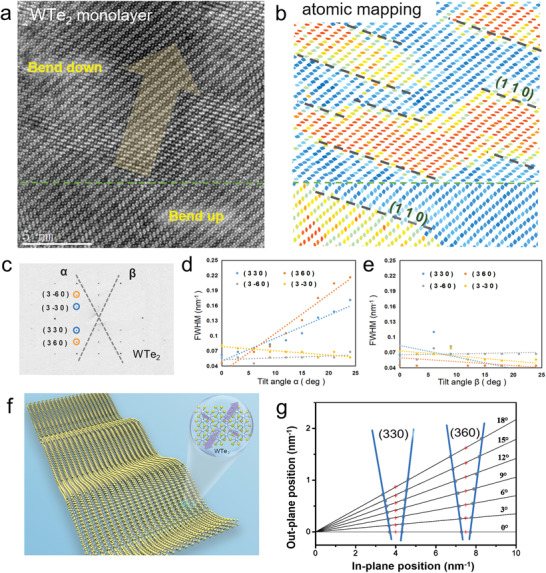
Anisotropy of continuous ripple in large‐scale WTe_2_ monolayer. a,b) STEM image of ripples propagating perpendicular to the (110) crystal planes and the corresponding spatial distribution mapping of the distorted quadrilateral units. The bending directions of ripple are determined by the distorted quadrilateral units, labeled as “Bend up” and “Bend down” in (a). The two bending sections are separated by green dotted lines. The boundary of the quadrilateral units in (b) shows that all ripples propagate perpendicular to (110) direction. c) TEM selected area electron diffraction (SAED) pattern of 1T’ WTe_2_ monolayer without tilting. DPs of (330) and (3−30) are marked by blue circles; (360) and (3−60) by orange circles. The tilt‐axis *α* and *β* are represented by grey dashed lines. d,e) FWHM of the intensity of four DPs in free‐standing monolayer WTe_2_ as a function of tilt angle d) *α* and e) *β* ranging from 0° to 25°. Dashed lines are the linear fits yielding the average roughness. The asymmetric FWHM broadening of (330)/(360) versus (3–30)/(3–60) along *α* and *β* axis suggests an anisotropic broadening of the corresponding relrods in the reciprocal space. f) Schematic diagram of anisotropic ripples in free‐standing WTe_2_ monolayer. The atomic structures are shown in insets, where different arrow size represents the fluctuation degree of ripple structure in that direction. g) The evolution of DPs with tilt angle *α* in monolayer WTe_2_. Each angle corresponds to a black solid line. For each tilt angle, the blue solid line represents a cross‐section for relrods of (330) and (360) in the reciprocal space.

The fine distribution of undulating structures in continuous large‐scale WTe_2_ monolayer is resolved by applying spatial mapping of the distorted quadrilateral units. **Figure** **3**a shows a large‐scale atomic STEM image with the corresponding mapping of the quadrilateral units (Figure 3b), indicating all the bending regions propagate perpendicular to (110) direction. Furthermore, biaxial tilted series of TEM diffraction imaging are designed to examine the ripple morphology anisotropy in a much larger scale of a continuous monolayer WTe_2_^[^ ^48^, ^49^ ^]^ (Figure S8, Supporting Information). Figure 3c shows a typical TEM selected area electron diffraction (SAED) pattern of monolayer WTe_2_ at 0˚ tilting, revealing its low symmetric unit cell structure without rotational symmetry. The incident angle of the TEM electron beam was changed by tilting the *α* and *β* axis of the TEM double‐tilt holder. As shown in Figure 3d, the full width at half maximum (FWHM) of the (330) and (360) DPswidens greatly as angle *α* increases, indicating that the corresponding reciprocal relrods are substantially broadened. This suggests that the atoms located on the crystal plane of (110) and (120) are collectively deformed due to the undulating structure (see more details in Figure S9, Supporting Information). However, the DPs of (3–30) and (3–60) remains almost unchanged as the *β* tilt angle increases (Figure 3e). The absence of DP widening in crystal planes of (1–10) and (1–20) when tilted along both *α* and *β* axis suggests the atoms located on the two crystal planes preserve nearly constant crystal plane spacing during titling, i.e., no undulating structure exists at such direction. Such asymmetric broadening during biaxial tilting can occur either in (110), (1–10), or (100) spot in different cases (see Figure S10, Supporting Information, for more detail), whereas cannot coexist in the same flake. Together with the atomic resolution STEM images of the intrinsic distortions of the lattice, we confirm that the ripple distribution in free‐standing WTe_2_ monolayer is anisotropic, as shown by the schematic in Figure 3f, which is completely different from the isotropic corrugation in other 2D materials (see Figure S11, Supporting Information, for the biaxial tilted TEM diffractions on MoS_2_ monolayer) reported previously.^[^ ^48^, ^50^ ^]^

For monolayer WTe_2_ membranes, the cone angles of relrods are valued between 12° and 16° as shown in Figure 3g. The lateral length *L* of the corrugations can be directly estimated as ≈12 nm from large‐scale HAADF‐STEM images. Therefore, the average height of anisotropic ripples in 1T’ WTe_2_ monolayer surface is calculated as ≈2.5 nm, which can also be confirmed clearly by AFM measurements of the free‐standing WTe_2_ monolayer on TEM grid (Figure S12, Supporting Information). Such large out‐of‐plane rippling may originate from the anisotropic nature, leading to concentrated deformation along certain planes induced by thermal perturbation. The formation of anisotropic ripple with exclusive propagation axis in monolayer WTe_2_ originates from the low symmetric lattice (1T’ phase) with anisotropic W‐Te bonding, which is further elaborated by DFT in the energy landscape (Figures S13 and S14, Supporting Information).

**Figure 4:**
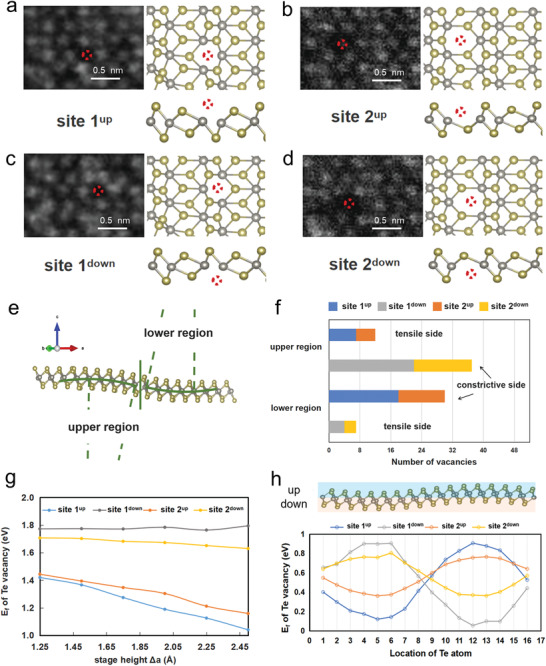
Anisotropic distribution of intrinsic Te vacancies modulated by ripple structure in WTe_2_ monolayer. a–d) HAADF‐STEM images and the atomic structure of four distinct Te vacancy sites in WTe_2_ monolayer. Red dotted circles represent the positions of the missing Te atoms. e) Atomic structure of the bending region from the side view. The bending is divided into the upper and lower regions, which are symmetric according to the change of the slope. The bending trend of W atom chain is depicted by green semicircles, including constrictive and tensile sides. f) Statistical counting of the four Te vacancy sites in the upper and lower deformation regions. Te vacancies are distributed preferentially on the constrictive parts. g) Calculated formation energy of the four distinct Te vacancy sites in the lower region as a function of the bending degree. Te vacancies located in the upper constrictive layer (site 1^up^ and site 2^up^) maintain a lower formation energy than that in the tensile layer (site 1^down^ and site 2^down^). h) Ripple‐induced modulated formation energy of the four Te vacancies in the supercell of ripple. Chalcogen atoms located at top and bottom layers are represented by blue and orange shades, respectively. The formation energy of Te vacancies fluctuates along with the ripple morphology. For detail in the statistics, see the Statistical Analysis section.

Since only certain crystal planes are bended, the anisotropic structural deformation is more likely to induce selective strain across the lattice and form preferential defects. We then examined the distribution of intrinsic Te vacancy in corrugated monolayer WTe_2_. The anisotropic unit cell of 1T’‐WTe_2_ gives rise to four distinct Te vacancy sites: the two near W atom, namely site 1 (**Figure** **4**a,c), and the other two away from W atom, namely site 2 (Figure 4b,d). Each of these Te vacancy sites can locate up or down in the lattice, as labeled by the superscript index. Moreover, we divided the distorted rippling region into two deformed parts depending on the change of the slope, namely upper and lower regions, as shown in Figure 4e. Both of these regions contain the constrictive and tensile sides. Accordingly, the geometrical positions of the Te sites (like site 1^up^ vs site 1^down^) become important since they are not identical as in the flat case.

The statistical results of these four kinds of Te vacancies in the ripple region reveals completely different distribution scenario from the previous theory.^[^ ^51^ ^]^ Figure 4f shows that Te vacancies located preferentially at the constrictive sides of the bending, regardless of their anisotropic lattice sites. For instance, Te vacancy site 1^down^ and site 2^down^ which are at the constrictive side overwhelm the other two in the upper region, while significantly being surpassed when switched to the tensile side in the lower region. Similar behavior is found for the Te vacancy site 1^up^ and site 2^up^. Based on the statistical analysis of the effect of mean spacing of Te atoms and ripple curvature on Te vacancy distribution (Figure S15, Supporting Information), we speculated that shorter mean Te spacing and larger bending curvature give rise to stronger repulsive forces for Te atoms in the constrictive inner layer to against the adjacent binding, thus easier to escape and form vacancy defects. Therefore, the anisotropic ripple structure has substantially modulated the defects in monolayer WTe_2_, in which the morphology induced deformation overwhelms the anisotropic lattice bonding in the formation of Te vacancy.

DFT is used to further understand such ripple‐induced preferential formation of Te vacancy. We first calculated the formation energy of the four distinct Te vacancy sites in the case of lower region (upper region is similar), as shown in Figure 4g. The results show that Te vacancies located at the constrictive side (site 1^up^ and site 2^up^) exhibit a lower formation energy than those at the tensile layer (site 1^down^ and site 2^down^), due to the asymmetric strain distribution which is much larger at the constrictive area. Moreover, the formation energy of the constrictive Te vacancy even goes lower if the bending deformation becomes larger, which suggests that as the ripple becomes intense, Te vacancy would preferentially accumulate at the constrictive Te layer. Furthermore, it can be clearly seen that the formation energy of all Te vacancy sites fluctuates depending on their local morphology (Figure 4f), consistent with our local strain analysis (Figure S15, Supporting Information), whereas reaching the lowest at the most constrictive site. It is notable that the anisotropic lattice bonding still plays a role in affecting the defect formation, in which the vacancy formation energy of site 1 fluctuates much severer than site 2. This is due to the closer W‐Te bonding of site 1, making it more sensitive to the ripple‐modulated strain. Moreover, the electronic structure of the anisotropic ripple is also calculated. WTe_2_ is semimetal but becomes semiconducting if considered the SOC effect. The SOC induced gap is calculated as 0.22 eV by DFT, slightly larger than that of the flat case (0.18 eV), as shown in Figure S16 in the Supporting Information, which is consistent with the previous work.^[^ ^52^ ^]^ It is expected that such SOC induced gap can be further regulated by modifying the ripple morphology.

**Figure 5:**
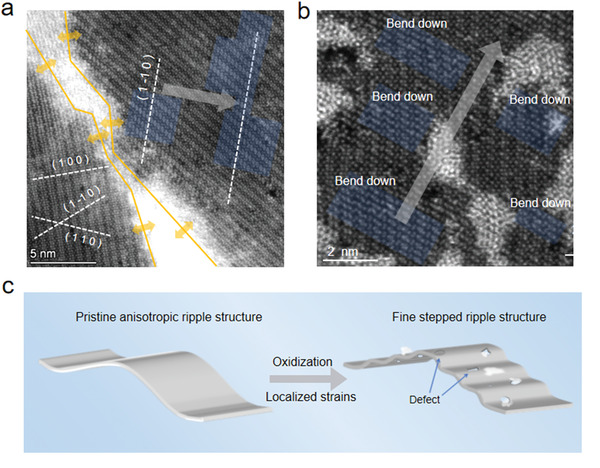
Anisotropic response of the ripple to the differently oriented grains and structure in partially oxidized case. a) STEM image of ripples near grain boundary (GB) in monolayer WTe_2_. Yellow arrows indicate the possible strain direction from the GB. The crystal plane of (100), (110), and (1−10) on both sides are marked by white dashed lines. The direction of the ripple in the right grain is indicated by the gray arrow. b) Atomic‐scale STEM image of fine step‐like ripple structures within partially oxidized WTe_2_ monolayer. Fine step‐like bending regions are shaded in blue. c) Schematic diagram of the transition process from a continuous smooth ripple structure to fine step‐like structure in WTe_2_ monolayer induced by oxidization.

Since the anisotropic ripple in free‐standing WTe_2_ monolayer propagates perpendicular to only one preferential crystal plane direction, it is straightforward to imply that such preferential bending axis depends on the initial strain applied from the boundary or edge. This can be verified in the occurrence of ripple near the grain boundary (GB), since different grains with diversified orientations lead to different response to the strain from the GB, as shown in **Figure** **5**a. Anisotropic distorted rippling structure along the direction perpendicular to the (1−10) crystal plane has been discovered only at the right side of the grain boundary; while no ripple pattern has been found at the left side. This is because the direction of the strain from the GB (yellow arrow) is perpendicular to the (1–10) crystal plane in the right grain, whereas nearly parallel to the planes of (100), (1−10), and (110) in the left grain. This suggests that, when strain coming in the preferential directions during the two grains merged and form GB, the atoms sited in that direction are prone to be shifted to form anisotropic ripple, which serves as one of the underlying sources of anisotropic ripple formation in WTe_2_ monolayer. Moreover, it is found that when monolayer WTe_2_ is partially oxidized, continuous ripple would induce several successive step‐like structures (Figure 5b,c). By measuring the directions of the squashed quadrilateral units, all the distorted regions in Figure 5b are determined to be bending along the same out‐of‐plane direction (bend down in this case). Such phenomenon implies that the anisotropic ripple structure could be modulated by localized strain from defects and oxidized nanoclusters, which softens the continuous ripple into several fine step‐like structure within the lattice. This process is in favor of releasing strain accumulated in the oxidized monolayer lattice which stabilizes the anisotropic rippling structure in monolayer WTe_2_.

## 3 Conclusion

In conclusion, by isolating the entire process including growth, transfer and atomic STEM characterization from air, we directly observe the large‐scale intact lattice structure of air‐sensitive 1T’‐WTe_2_ monolayer. The collective lattice distortion is visualized, due to the presence of anisotropic rippling, with a dominating propagation direction perpendicular to the crystal plane of either (100), (110) or (1–10). The direction and fluctuation trend of ripples can be identified by the distorted unit in the projection. The formation of ripple anisotropy is due to the lack of rotational symmetry in 1T’ structure, whereas the WTe_2_ monolayer exhibits axis‐dependent response and tolerance to the strain. Moreover, Te atoms tend to escape away from the constrictive side of the undulating ripple structure, exhibiting the intrinsic anisotropy of defect distribution. Our work inspires a new understanding of the structural stability in WTe_2_ monolayer with low symmetry, pave the way for defect engineering for tailoring the properties in emerging air‐sensitive 2D materials.

## 4 Experimental Section

### CVD Growth

Metal precursors which consist of 15 mg NaCl and 60 mg WO_3_ were located in the center of the heating zone, and Te powder was placed at upstream away from heating center of 9 cm. The furnace was heated to 820 °C in 16 min and held at this temperature for 5 min before cooling down to room temperature. 80 sccm Ar and 20 sccm H_2_ acted as carrier gas during the growth process. After cooling down, the sample was then fetched from the furnace to the glove box directly through a connecting chamber.

### Sample Preparation

An isopropanol assisted direct transfer method was used for the preparation of STEM samples. A TEM grid (Quantifoil Au grid) was placed on m‐plane quartz wafer with WTe_2_ monolayers samples. A drop of isopropanol was gently placed on top of the grid and bake at 75 °C for 15 min. Afterward, the wafer was immersed in HF‐H_2_O (1:4 by vol) solution to etch away the silica coating. After lifting off, the Au grid was transferred to deionized water for several cycles to wash away the residual contaminations and dried in the inert gas atmosphere. All transfer processing were performed inside the specially designed interconnected glove‐box system to avoid any sample degradation.

### TEM‐DF and STEM Characterizations

Electron diffraction patterns were carried out on an FEI Tecnai 20T operated at 80 kV. No significant damage was observed on the WTe_2_ monolayers under this voltage. The TEM‐DP images were recorded using specific diffraction spots selected by the objective aperture. The atomic‐scale HAADF‐STEM images were acquired from an aberration‐corrected scanning transmission electron microscope (FEI Tian Themis 60–300 kV, operate at 60 kV). Cs double corrector DCOR (CEOS) and a high‐brightness field emission gun (X‐FEG) with monochromator were assembled in this TEM for better image quality. The inner and outer collection angles for the STEM images (*β*1 and *β*2) were 52 and 200 mrad, respectively, with a semiconvergence angle of 30.1 mrad.

### DFT Calculation

Density functional theory calculation was performed using PWmat code which runs on graphics processing unit processors. Norm‐conserving SG15 pseudopotential was used with 50 Ryd cutoff energy in all the calculations. Perdew–Burke–Ernzerhof (PBE) exchange‐correlation functions were used in all calculations. Monkhorst‐Pack method^[^ ^34^ ^]^ was used when the *k* points sampling was done with the number of *k* points multiplied by the number of atoms were larger than 1000. A DFT‐D2 semiempirical dispersion‐correction approach was used to correct the van der Waals interactions.

The total energy calculation was performed with cell relaxation where the residual stress was less than 0.01 eV per atom and with atomic position relaxation where the residual force on each atom was less than 0.01 eV Å^−1^. To build the bended structure, the *z*‐positions of W atoms were fixed as the followingfunction

(1) $$\Delta z=d\times \sin\left(\frac{y}{b}\times 2\pi \right)$$

Here, Δ*z* is the fluctuation in *z* direction, *y* is the atomic position in *y* direction, *b* is the unit cell length in *y* direction. In the total energy calculation of curved structure, the *z* positions of W atoms were fixed whereas other atoms and the *x*, *y* position of W atoms can be fully relaxed. In the calculation of shortened *b* length cell, the cell parameters are fixed in the calculations. After relaxation calculation of WTe_2_, the force can be in *z*‐direction of each W atom. These forces were used in formation energy calculation of Te vacancy in WTe_2_, where each W atom was fully relaxed in *x*, *y*, and *z* direction with the external force in *z* direction calculated in previous steps. The formation energy of Te vacancy was used in the following formula

(2) $$E_{\mathrm{Te}\;\mathrm{vacancy}}=E_{\mathrm{Te}}+E_{W_{32}Te_{63}}-32E_{\mathrm{WT}e_{2}}$$

Here, *E*_Te_ is the total energy per atom of trigonal Te simple substance. And $E_{W_{32}Te_{63}}$ is the total energy of a WTe_2_ with 1/64 Te vacancy.

### Statistical Analysis

Distortion Mapping of Ripple Area: The 2D projection coordinates of atoms were derived from HAADF‐STEM images by using Calatom software,^[^ ^53^ ^]^ before which the nonuniform background was eliminated and a block‐matching and 3D filtering algorithm (BM3D) filter method were used. The W/Te atomic distance in a quadrilateral unit cell can be depicted based on the 2D projection coordinate data, which represents the degree of lattice distortion (as shown in Figure 3b).

Statistics of Te Vacancy Distribution: High‐resolution atomic images from multiple flakes were used for defect statistics. The Te vacancies in WTe_2_ monolayer were classified into four distinct sites. Only single Te vacancies were counted in the statistical analysis presented in Figure 4f. To ensure the reliability of the data, the Te vacancies distribution was performed on multiple sets of experimental data of around 1000 nm^2^. A value of *P* < 0.05 was considered statistically significant.

## Conflict of Interest

The authors declare no conflict of interest.

## Author Contributions

K.N. and M.W. contributed equally to this work. J.L. conceived the project. K.N. made the TEM samples, performed AFM measurement, TEM‐DP and STEM related experiments, analysis, and simulations. Z.G. and S.L. grew monolayer WTe_2_ samples. M.H. and G.W. participated in parts of the STEM characterizations. DFT calculations were done by M.W. and F.P. The paper was written by K.N. and J.L. with input from all authors. All authors commented on the paper.

## Supporting information

Supporting Information

## Data Availability

Research data are not shared.
