# Supplementary material for: Direct Visualization of Large‐Scale Intrinsic Atomic Lattice Structure and Its Collective Anisotropy in Air‐Sensitive Monolayer 1T’‐ WTe2
Source: Adv Sci (Weinh). 2021 Aug 31;8(20):2101563. doi: 10.1002/advs.202101563 (PMC8529427; doi:10.1002/advs.202101563)
Supplement: Supplementary file 1 — Supporting Information [file ADVS-8-2101563-s001.pdf]

© 2021 Wiley-VCH GmbH

ADVANCED SCIENCE
Open Access

Supporting Information

for *Adv. Sci.,* DOI: 10.1002/advs.202101563

Direct visualization of large-scale intrinsic atomic lattice structure and its collective anisotropy in air-sensitive monolayer 1T'- WTe_2

*Kangdi Niu, Mouyi Weng, Songge Li, Zenglong Guo, Gang Wang, Mengjiao Han, Feng Pan**, and *Junhao Lin**

Supplementary Materials for:

Direct visualization of large-scale intrinsic atomic lattice structure and its collective anisotropy in air-sensitive monolayer 1T'- WTe_2

Kangdi Niu[1,3‡], Mouyi Weng[2‡], Songge Li[1], Zenglong Guo[1], Gang Wang[1], Mengjiao Han[1,3], Feng Pan[2], Junhao Lin[1,3*]*

[1]Department of Physics, Southern University of Science and Technology, Shenzhen 518055, China

[2] School of Advanced Materials, Peking University, Shenzhen Graduate School, Shenzhen 518055, China

[3]Shenzhen Key Laboratory of for Advanced Quantum Functional Materials and Devices, Southern University of Science and Technology, Shenzhen 518055, China

[‡]These authors contributed equally to this work.

[*]Corresponding author. E-mail: linjh@sustech.edu.cn; panfeng@pkus.edu.cn

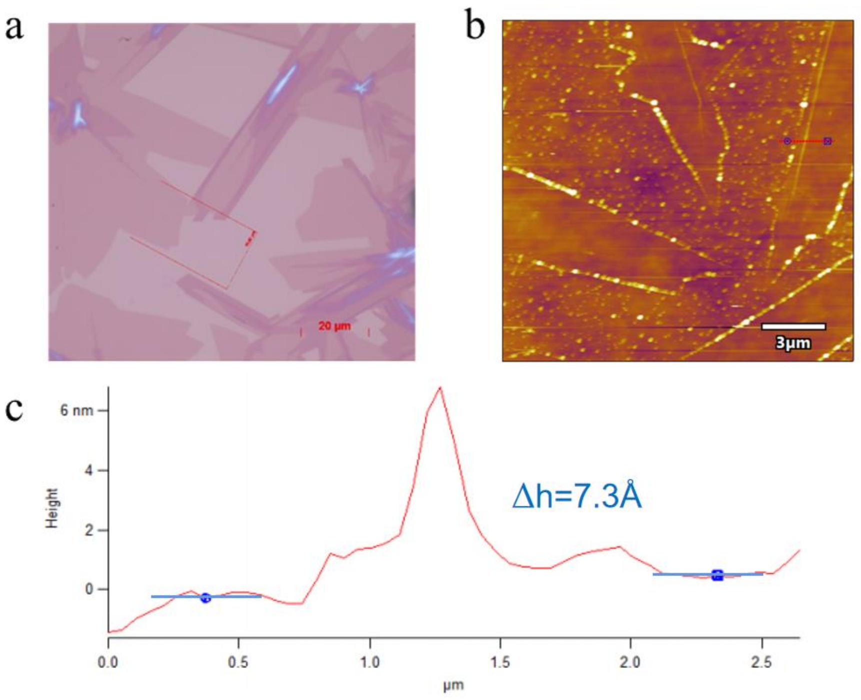

Figure S1. Optical microscopy (a) and atomic force microscopy (AFM) (b, c) characterizations of WTe_2 monolayer. The optical contrast and thickness measurement confirm that most of our CVD-grown WTe_2 flakes are monolayer.

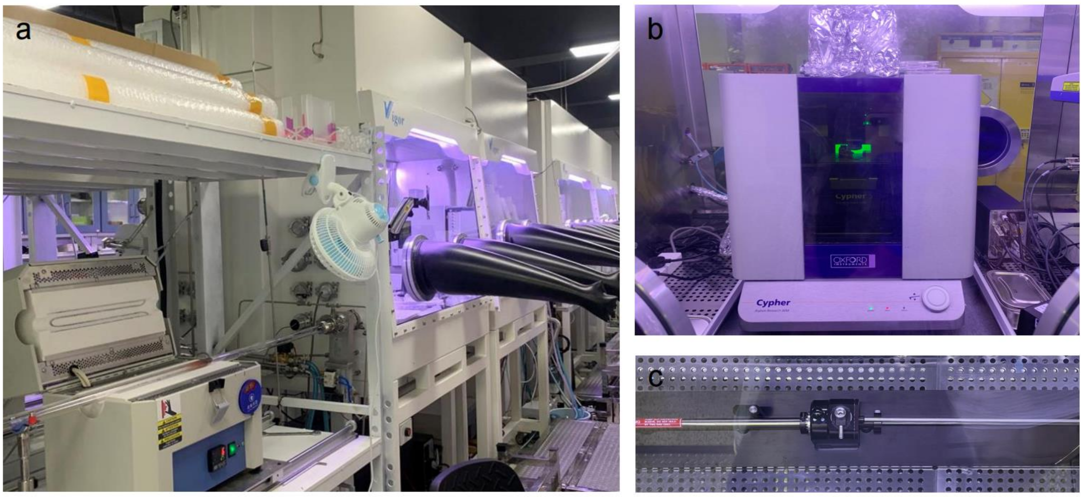

Figure S2. Home built glovebox connected system. (a) Chemical vapor deposition (CVD) tube furnace connected to the glove box. (b) Atomic force microscope (AFM) mounted in glove box. (c) Vacuum transfer holder. The glovebox connected system provides water-oxygen isolation environment for the entire growth, transfer and characterization process of WTe_2 monolayer to ensure its structural integrity.

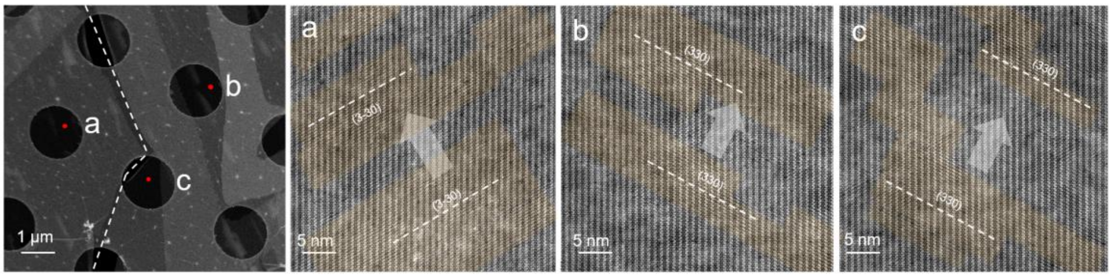

Figure S3. STEM characterization of different regions in CVD-grown WTe_2 monolayer. Distorted regions are shaded in orange. Arrows indicate the undulating direction of the ripples. The crystal plane of (3-30) and (330) are marked by white dotted lines.

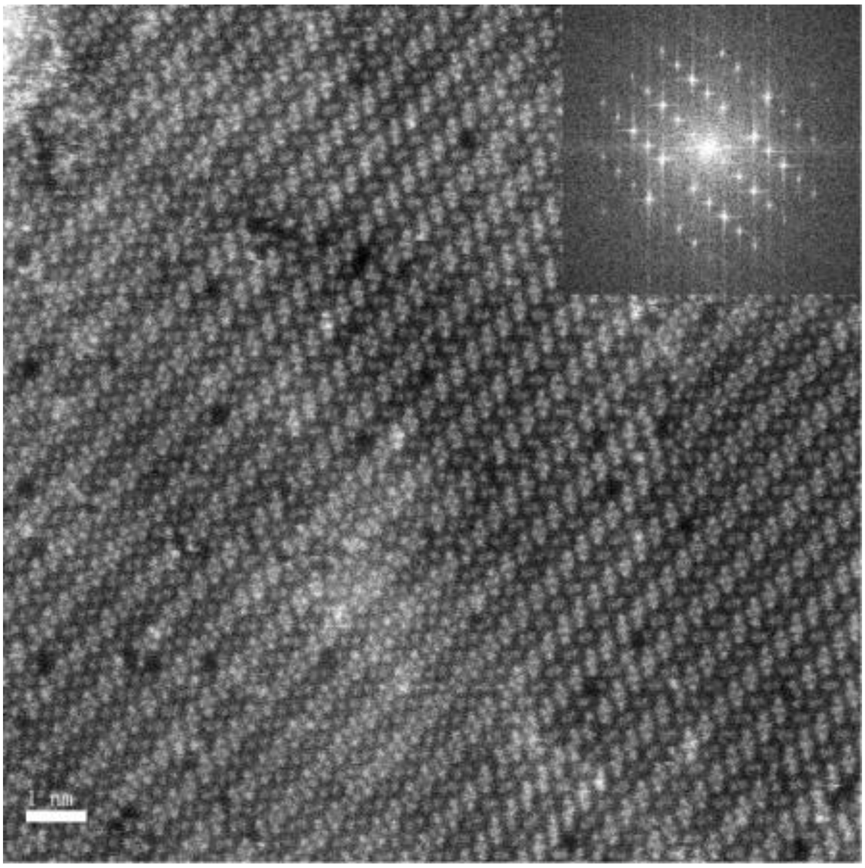

Figure S4. A wide range of ripple structure within WTe_2 monolayer. The inset illustration shows Fast fourier transformation (FFT) of the ripple structures without additional symmetry.

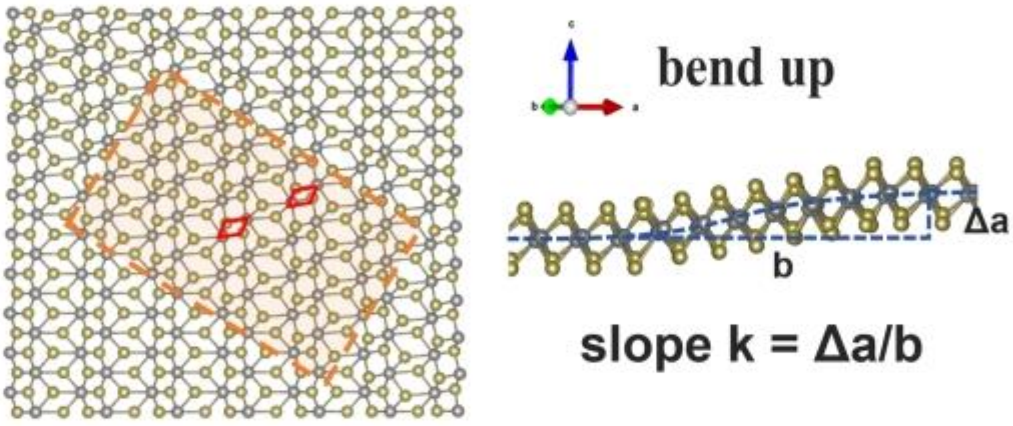

Figure S5. Atomic ripple structure of WTe_2 monolayer. The bending slope of ripple structure in this case is calculated as ~ 0.2.

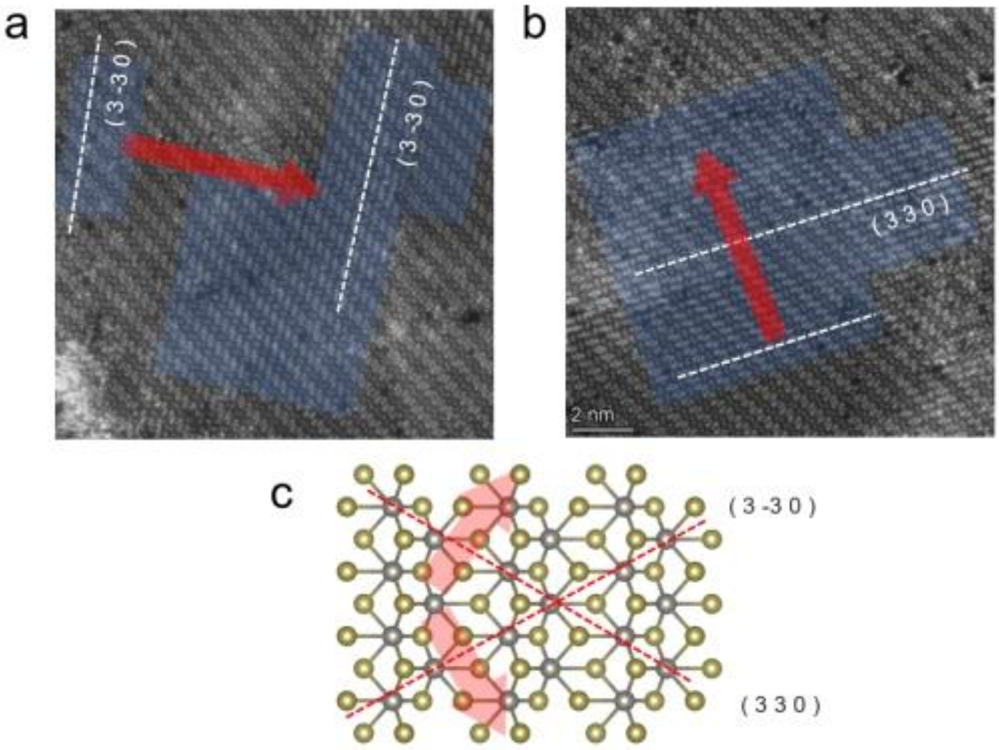

Figure S6. Anisotropic ripple structures in different directions. (a) and (b) are HAADF-STEM images of the WTe_2 ripples perpendicular to the direction of the (1-10) and (110) crystal plane (equivalent to (330) and (3-30) crystal planes), respectively. Red arrows indicate the undulating direction of the ripples. The crystal plane of (1-10) and (110) are marked by white dotted lines. (c) Atomic structure of WTe_2 monolayer showing the symmetrical lattice planes of (1-10) and (110). Ripples selectively occur in directions perpendicular to either (110) or (1-10) crystal plane.

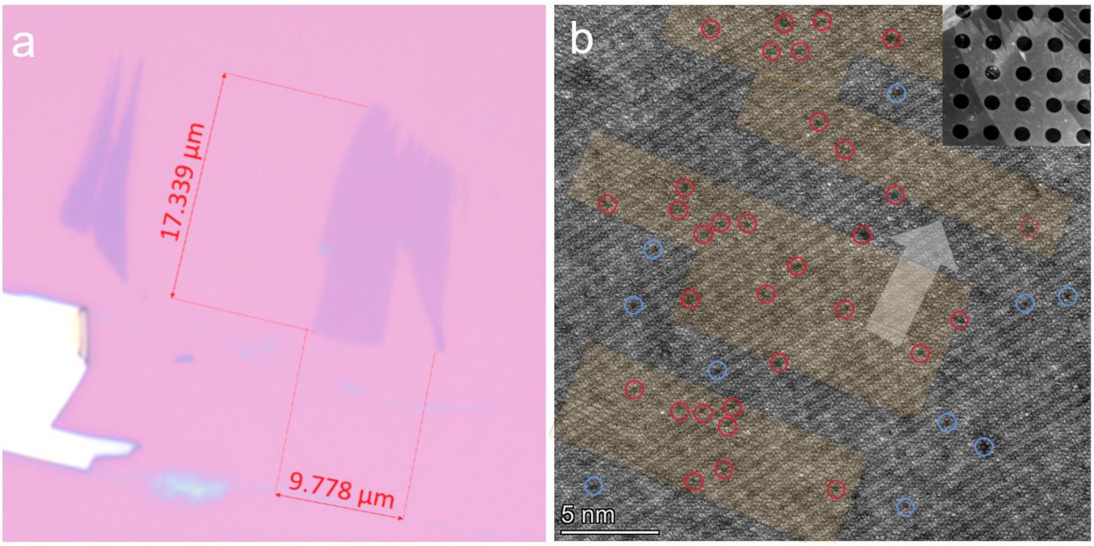

Figure S7. Atomic-scale HAADF-STEM images of the mechanical exfoliated WTe_2 monolayer. Distorted regions are shaded in orange. Arrows indicate the undulating direction of the ripples. Red circles indicate the Te vacancies in the distorted region, and the blue circles indicate those in the flat region. It is obvious that vacancies gather in the distorted rippling area, which is consistent with the statistical distribution of Te vacancies obtained from CVD-grown monolayers in the main text.

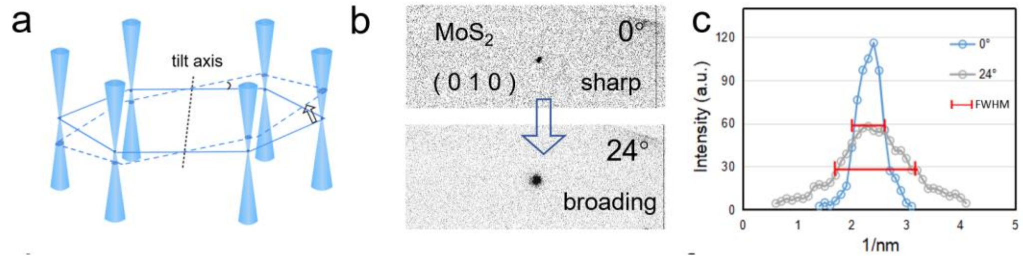

Figure S8. (a) The schematic reciprocal space for a corrugated monolayer with hexagonal lattice, where the tangent positions between the Ewald sphere and the relrods are marked by blue circles. (b) TEM diffraction peak of (010) in MoS_2 monolayer under 0° and 24° tilted angles. (c) The intensity of DPs in (b). The full width at half maxima (FWHM) is widened as the tilted angle increases.

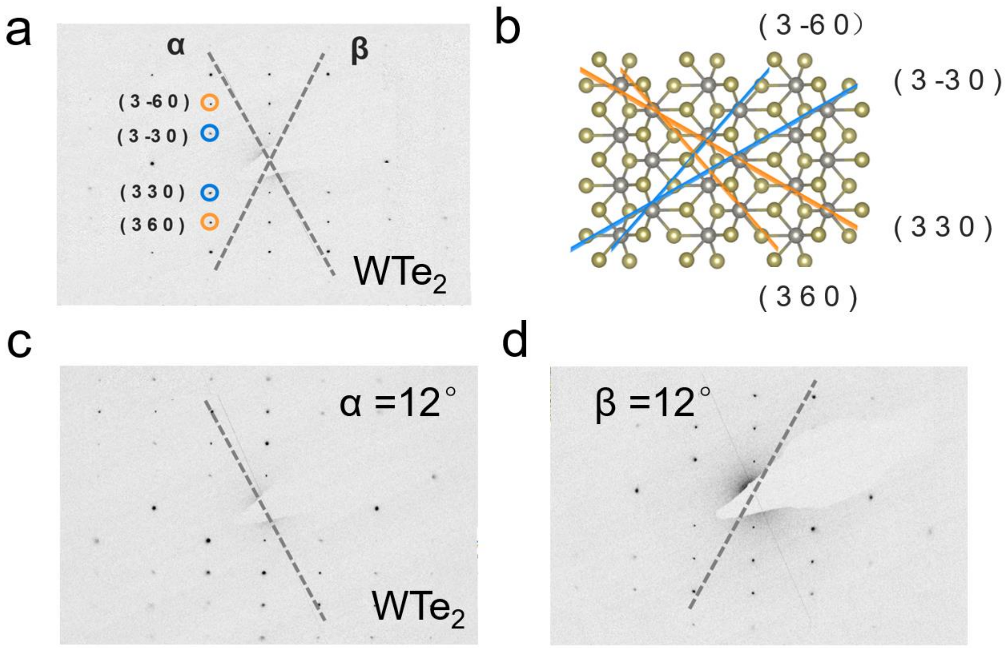

Figure S9. TEM diffraction patterns and the corresponding crystal planes in WTe_2 monolayer. (a) are TEM DPs of WTe_2 monolayer without tilting shown in main text. (b) are the atomic structure of WTe_2 in real space with the corresponding crystal planes represented by corresponding blue and orange lines. (c) and (d) are TEM DPs of WTe_2 under incidence angles of 12° along axis α and axis β, respectively.

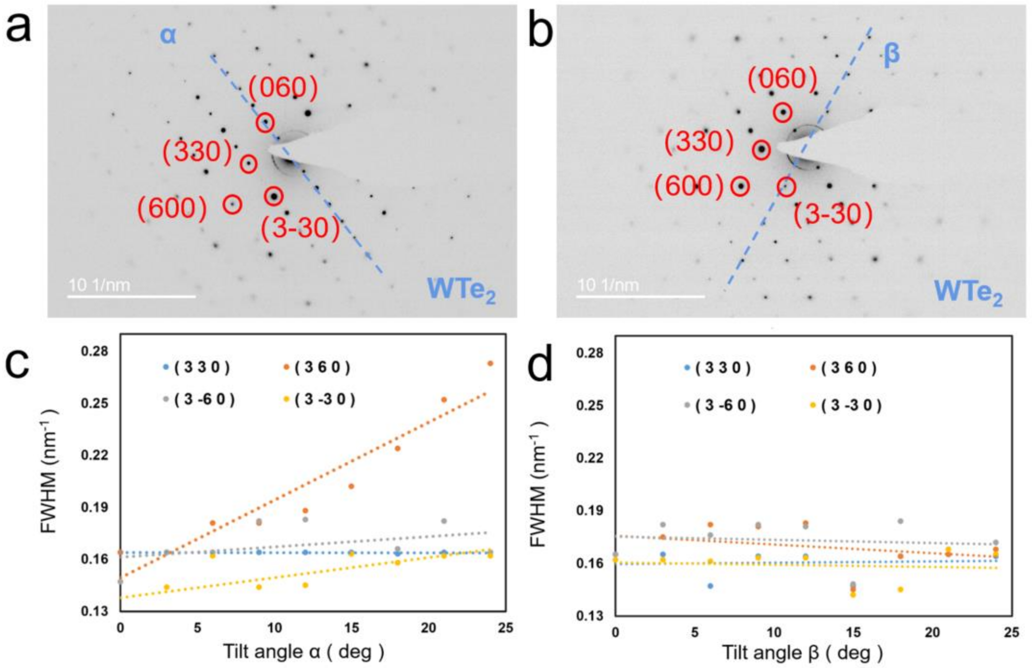

Figure S10. Tilted TEM-DP experiments of free-standing 1T' WTe_2 monolayer. (a, b) TEM selected area electron diffraction (SAED) pattern of under incidence angles of 21°along axis α and axis β, respectively. DPs of (330), (3-30), (060) and (600) are marked by red circles; (c, d) FWHM of the intensity of three DPs in free-standing monolayer WTe_2 as a function of tilt angle α (c) and β (d) ranging from 0° to 25°. Dashed lines are the linear fits yielding the average roughness. DPs widening in crystal planes of (110) and (010) is absent when tilted along two axes, *i.e.*, little structural deformation in the (110) and (010) crystal planes but overwhelms in (100), which suggests the ripples occur in the direction perpendicular to the (600)/ (100) crystal plane. Note that theory suggest that the formation energy among (100), (010) and (110) (or (1-10)) is small, so, in addition to the two kinds of ripple structures observed here, it is speculated that ripple propagating perpendicular to the (010) direction could also exist, which may be observed in future studies.

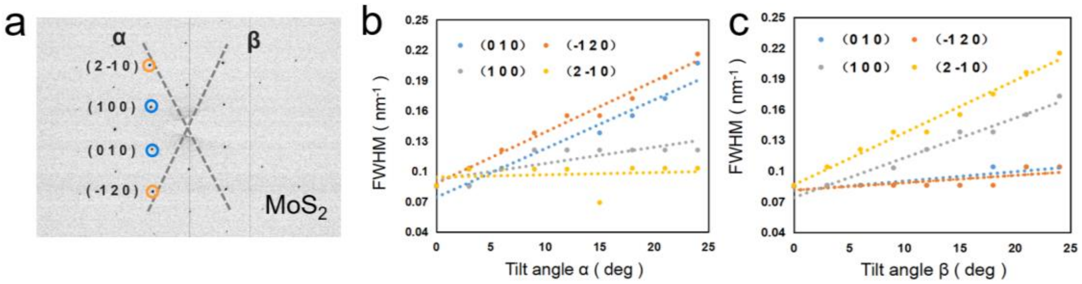

Figure S11. Tilted TEM-DP experiments of free-standing 2H MoS_2 monolayer. (a) SAED pattern of 2H MoS_2 monolayer without tilting, and evolution of intensity FWHM in free-standing monolayer MoS_2 tilted along α (b) and β (c) axis, respectively. The FWHM widening of equivalent DPs (α: 010 versus β: 100) is similar as tilted along two axes, suggesting the ripple distribution in MoS_2 monolayer is isotropic.

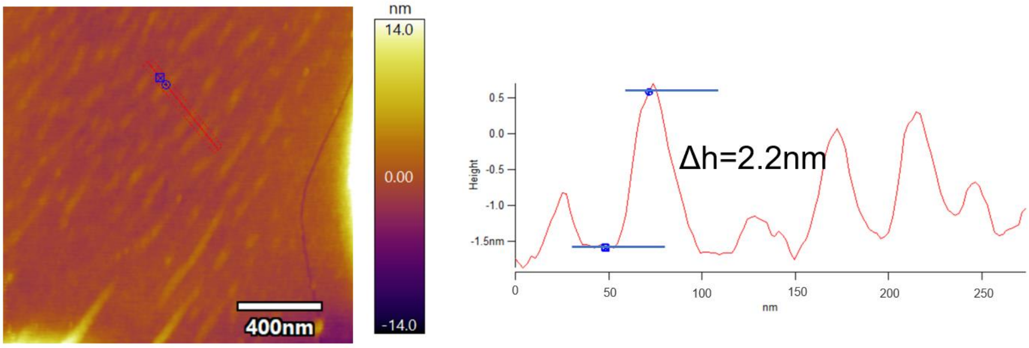

Figure S12. AFM characterizations of WTe_2 monolayer on Quantifoil Au grid. The height of the ripple supported by the carbon film is accurately measured as 2.2 nm.

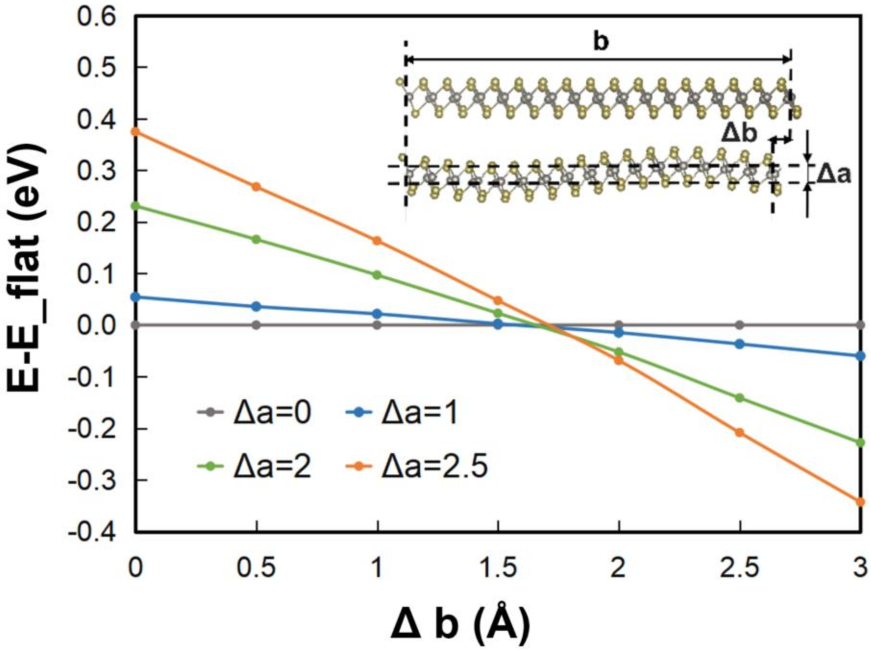

Figure S13. Calculated formation energy difference of corrugated and flat WTe_2 monolayer flakes. The vertical and horizontal variations of WTe_2 flake are denoted by Δa and Δb. WTe_2 flat monolayer tends to be deformed as an out-of-plane ripple structure when Δb is greater than 1.7 Å. The energy difference becomes larger as the compression increases, suggesting it is easier to release the accumulated strain by inducing out-of-plane deformation instead of shortening the bonds in-plane.

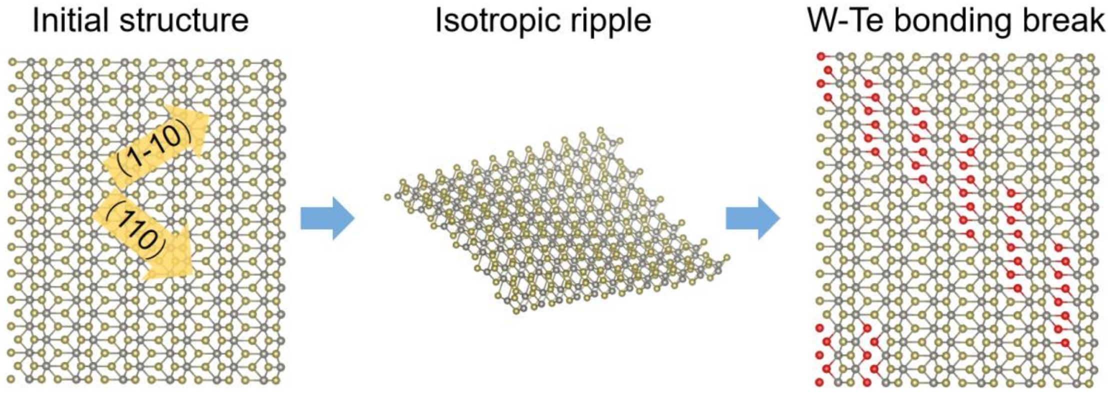

Figure S14. Atomic model of 1T' WTe_2 monolayer with isotropic ripple. This bending structure in low symmetric will result in continuous elongation of certain bonding between W and Te atoms (labeled red), which can be stretched as long as 3.2 Å. Such W-Te bonds are irrational, resulting in an unstable structure and collapse of the monolayer, consistent with TEM and STEM data that ripples along two feasible directions cannot co-exist in the same region of WTe_2 monolayer.

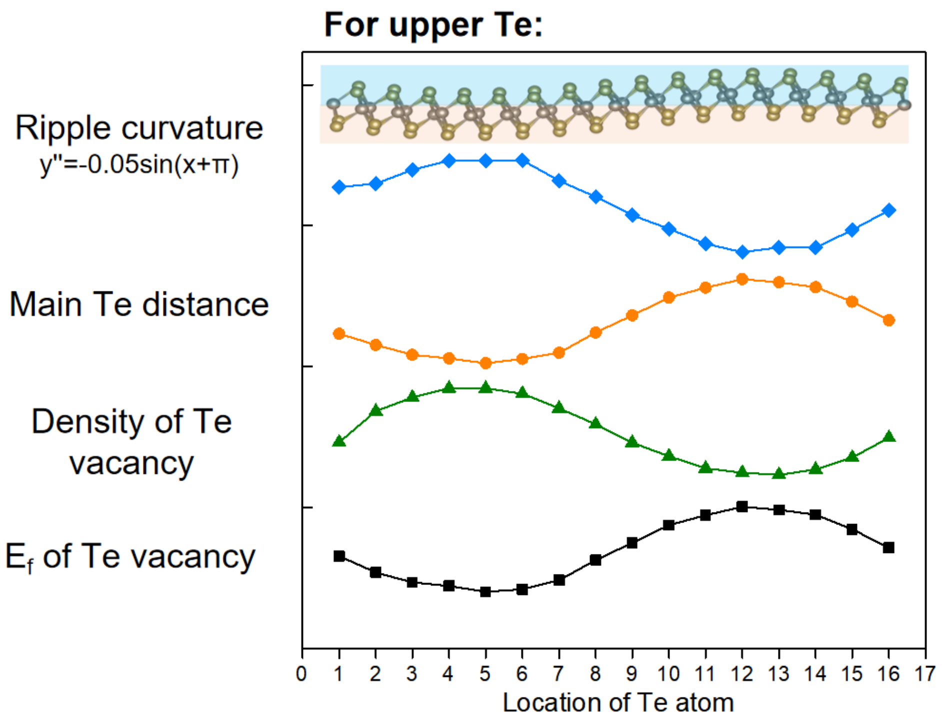

Figure S15. The effect of mean distance of Te atoms and ripple curvature on Te vacancy distribution**.**

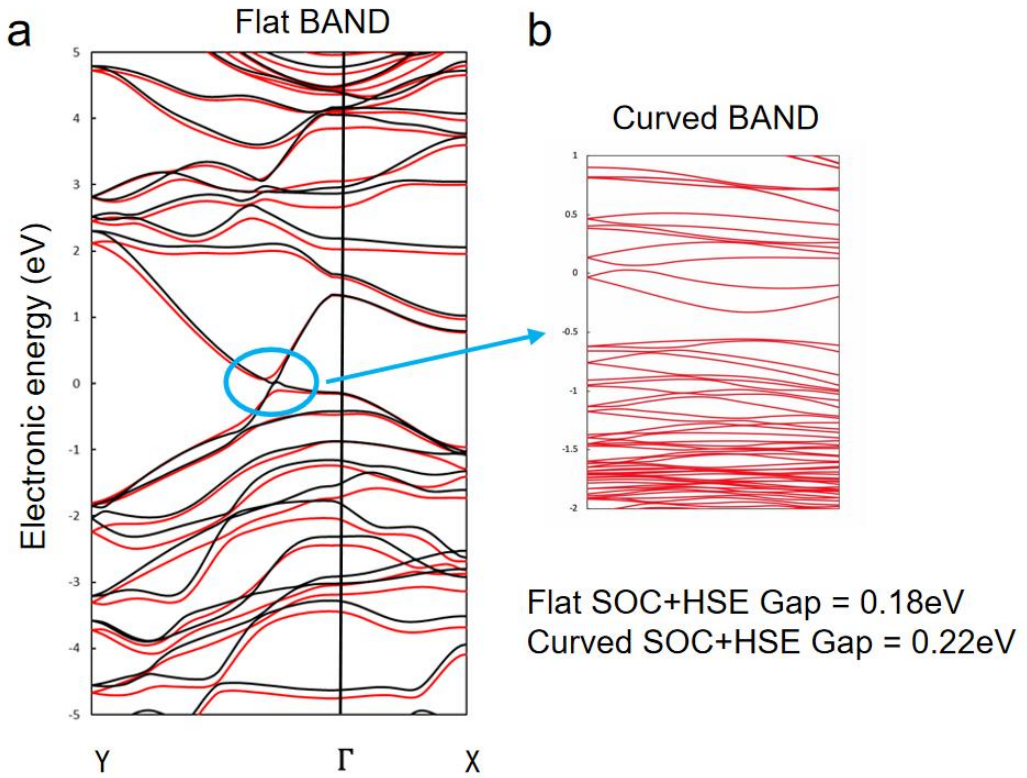

Figure S16. Band structure of 1T' WTe_2 monolayer. (a) Band structure of flat WTe_2. Black line is calculated by PBE, red line is calculated by HSE+SOC. (b) Band structure of curved WTe_2. Because curved structure is calculated in supercell, band structure is folded in (b).
